# Effects of balloon pulmonary angioplasty on cardiac and pulmonary functions and complications in patients with chronic thromboembolic pulmonary hypertension

**DOI:** 10.3389/fmed.2025.1689193

**Published:** 2026-01-12

**Authors:** Heng Wang, Mingzhe Cui

**Affiliations:** Department of Vascular Surgery, Henan Provincial People’s Hospital, Zhengzhou, Henan, China

**Keywords:** balloon pulmonary angioplasty, cardiac function, chronic thromboembolic pulmonary hypertension, Hemodynamics, pulmonary function

## Abstract

**Objective:**

To explore the effects of balloon pulmonary angioplasty (BPA) on cardiac and pulmonary functions and complications in patients with chronic thromboembolic pulmonary hypertension (CTEPH).

**Methods:**

From February 2023 to December 2024, 100 patients with CTEPH at our hospital underwent BPA treatment were selected. The WHO cardiac function classification, right heart catheterization parameters, cardiac function indexes, levels of NT-proBNP, TNF-*α* and IL-6, exercise tolerance, pulmonary function indexes, pulmonary artery pressure, and blood gas indexes were compared before BPA and 3 months after the last BPA. The BPA-related complications were recorded.

**Results:**

Compared with before BPA, the WHO cardiac function classification of Grade I, Grade II and Grade III was significantly improved 3 months after the last BPA (*p* = 0.003, *p* = 0.020, *p* = 0.000), the mPAP and PVR were significantly lower (*p* < 0.001), the CI was significantly higher (*p* < 0.001), the TAPSE, RVFAC and EDV were significantly higher (*p* < 0.001), the TVR was significantly lower (*p* < 0.001), the 6-MWD was significantly longer (*p* < 0.001), the FVC, FEV1 and FEV1/FVC were significantly higher (*p* < 0.001), the levels of pulmonary artery systolic pressure and pulmonary artery diastolic pressure were significantly lower (*p* < 0.001), the levels of NT-proBNP, TNF-*α* and IL-6 were significantly lower, the PaO_2_ level was significantly higher (*p* < 0.001), as well as the PaCO_2_ level was significantly lower (*p* < 0.001). However, there were no significant differences in ESV and LVEF between before BPA and 3 months after the last BPA (*p* > 0.05). In addition, there were 5 cases of hemoptysis, 1 case of reperfusion pulmonary edema, and no other complications occurred.

**Conclusion:**

BPA can improve the exercise tolerance, cardiac and pulmonary functions, and blood gas level of patients with CTEPH, and the incidence of complications is relatively low. However, due to the limitations of the single-arm design, the results of this study need to be further verified through randomized controlled trials.

## Introduction

Chronic thromboembolic pulmonary hypertension (CTEPH) is characterized by the organization and fibrosis of blood clots that form after acute pulmonary embolism, resulting in narrowing of the pulmonary vessels and subsequent pulmonary vascular remodeling (1). It is a chronic thromboembolic disease that is accompanied by structural alterations in the pulmonary arteries (2). At the Fifth World Conference on Pulmonary Arterial Hypertension, CTEPH was classified as the fourth major type of pulmonary hypertension (3). The clinical symptoms of CTEPH are not typical, making it easily confused with other pulmonary and pulmonary vascular diseases, and it is prone to cause right heart failure (4). The prognosis of CTEPH is extremely poor, and its clinical management is challenging (5).

Currently, treatment options for CTEPH include surgical, drug and interventional therapies (6). Pulmonary endarterectomy (PEA) belongs to the preferred treatment for CTEPH (7). However, owing to the influence of comorbidities and lesion location, only 36.6% of patients are suitable for PEA (8), and 31% of CTEPH patients still have residual pulmonary hypertension after undergoing PEA (9). Additionally, PEA surgery is challenging and can only be performed in high-level cardiovascular medical centers. Studies have shown that targeted drugs, such as soluble guanylate cyclase stimulators and endothelin receptor antagonists can improve the hemodynamics and activity tolerance of CTEPH patients (10, 11); however, further research is required to confirm these findings.

In recent years, balloon pulmonary angioplasty (BPA) has become an alternative treatment option for CTEPH patients who cannot undergo PEA or have residual recurrence after PEA (12). In 2013, at the International Symposium on Pulmonary Hypertension held in Nice, and in the 2016 ERS/ESC Guidelines for Pulmonary Hypertension, it was proposed that BPA can be considered for patients who cannot undergo surgical treatment (13). The basic principle of BPA is to first perform pulmonary artery angiography using a catheter to locate narrowed or occluded pulmonary artery branches. Then, under the guidance of a guidewire, a balloon is used to expand the narrowed or occluded pulmonary artery branches to relieve the obstruction and lower the pulmonary artery pressure (14). Relevant studies conducted abroad have shown that the clinical symptoms and cardiac-pulmonary functions of patients with CTEPH after BPA treatment have significantly improved (15, 16). However, there are few reports on patients with CTEPH who have undergone BPA in China. This study aimed to evaluate the effects of BPA on cardiac and pulmonary functions, as well as complications in CTEPH patients.

## Materials and methods

### Patients

From February 2023 to December 2024, 100 patients with CTEPH at our hospital underwent BPA treatment were chosen to be study participants. Inclusion criteria: (1) Age ≥ 18 years; (2) Definite perfusion defect identified by lung ventilation/perfusion (V/Q) imaging; (3) Average pulmonary artery pressure measured by right heart floating catheter ≥ 25 mmHg, and pulmonary arteriolar occlusion pressure < 15 mmHg; (4) Cardiac function grades I to IV. Exclusion criteria: (1) Patients eligible for PEA surgery; (2) Creatinine clearance rate < 50 mL/min; (3) Transaminase levels more than 3 times the normal value; (4) Allergic to iodine contrast agents; (5) Active smokers; (6) Chronic lung diseases such as chronic obstructive pulmonary disease or interstitial lung disease. This study was approved by the ethics committee of our hospital and all patients signed the informed consent form.

### Sample size calculation

The initial sample size was estimated based on a pilot study (n = 20) in our center. Pre-experiment data showed that after BPA, the mean mPAP decreased by 14 ± 9 mmHg. To detect a difference of 14 mmHg (SD = 9 mmHg) with *α* = 0.05 and 1-*β* = 0.90, G*Power 3.1 suggested a sample size of 72 cases for a two-arm trial. Considering this was a single-arm study and allowing for a 20% dropout rate, we enrolled 100 patients to ensure sufficient power for subgroup analyses. A post-hoc analysis using the observed effect size (Cohen’s d = 1.20 for mPAP) confirmed that the actual statistical power (1-*β*) was close to 1, indicating the study was adequately powered to detect the effect of BPA on mPAP. However, this analysis was not used to justify the sample size *a priori*.

### Collection of clinical data

Clinical data were collected from the patients, which contained gender, age, body mass index (BMI), smoking history, drinking history, history of hypertension, history of diabetes, as well as the application of pulmonary artery targeted therapy and anticoagulant drugs.

Anticoagulant, thrombolytic, and pulmonary artery-targeted therapy details: (1) Anticoagulation: Low-molecular-weight heparin (LMWH, enoxaparin 1 mg/kg q12h) was initiated ≥3 days pre-BPA, transitioning to oral warfarin (target INR 2.0–3.0) or rivaroxaban (20 mg/d) post-BPA for 3 months. (2) Thrombolysis: Only acute/subacute CTEPH patients (duration <6 months) received urokinase (500,000 U/d IV for 5–7 days) or alteplase (50 mg IV over 2 h); chronic CTEPH patients received no thrombolytics. (3) Pulmonary artery-targeted therapy: All patients were treated with bosentan (62.5 mg bid, titrated to 125 mg bid) or sildenafil (25 mg tid) from diagnosis until 6 months post-last BPA. During BPA, heparinized saline (100 U/L) was used for catheter flushing to maintain ACT 250–300 s. Post-BPA anticoagulation adjustments were made at the discretion of treating physicians.

Preoperative treatment: Anticoagulation therapy is initiated at least 3 days before BPA surgery, and thrombolytic therapy (if applicable) is completed within 1 week before the surgery; targeted therapy is initiated immediately upon the diagnosis of CTEPH, without a fixed time interval from BPA surgery.

Intraoperative management: During the BPA procedure, heparinized normal saline (100 U/L) was continuously used to flush the catheter, maintaining the ACT (activated clotting time) within the range of 250–300 s.

Postoperative adjustments: If vascular injury or bleeding complications occur after BPA surgery, the anticoagulant drug dosage is temporarily reduced or suspended (for example, warfarin dosage is reduced to INR 1.5–2.0), and then restored after stabilization; the targeted treatment dosage was not adjusted due to BPA.

This study was a retrospective analysis. All patients followed the above standardized protocol and no treatment strategies were adjusted for the purpose of the study, so there were no “potential changes” in the treatment plans.

### BPA methods

Based on the severity of the patient’s condition, the complexity of the lesion, and the overall physical condition, the number of BPA sessions per patient ranged from 2 to 5, with a median of 3 sessions (IQR: 2–4). The decision on the total number of sessions was guided by the following criteria: (1) Lesion severity and distribution: Patients with diffuse, mild lesions (e.g., involvement of ≥3 pulmonary segments with mean pulmonary artery pressure (mPAP) ≤ 35 mmHg) typically received 3–5 sessions to achieve gradual dilation of multiple segments. In contrast, patients with localized, severe lesions (e.g., focal stenosis in central pulmonary arteries with mPAP ≥50 mmHg) underwent 2–3 sessions, focusing on critical segments to minimize procedural risks. (2) Hemodynamic response: After each session, patients underwent right heart catheterization to assess immediate hemodynamic improvements [e.g., reduction in mPAP and pulmonary vascular resistance (PVR)]. If mPAP decreased by <10% or PVR remained elevated (>5 Wood units), an additional session was planned to optimize therapeutic effects. (3) Comorbidities and procedural tolerance: Patients with poor vascular compliance (e.g., calcified lesions) or comorbidities (e.g., severe chronic obstructive pulmonary disease) received fewer sessions (2, 3) to avoid over-dilatation and vascular injury.

The interval between sessions was fixed at 4 weeks to allow for vascular remodeling and recovery. During each session, 1–3 lesion segments were treated, with prioritization given to segments with the highest pressure gradients or clinical significance. For patients with scattered lesions, up to 4 segments were occasionally treated in a single session after careful hemodynamic monitoring.

The patient underwent routine disinfection and bandaging procedures, and local anesthesia was administered at the puncture site using 1% lidocaine. A 70–90 cm 8F Cook vascular sheath (from the United States) or an 80 cm 8F ArrowSuper Flex vascular sheath (from the United States) was inserted through the femoral vein. Then, 50 U/kg of unfractionated heparin (UFH) was administered intravenously (reference dose range: 30–100 U/kg per the 2021 ESC/ERS Pulmonary Hypertension Guidelines; our center’s protocol prioritizes 50 U/kg to balance anticoagulation efficacy and bleeding risk in CTEPH patients with moderate-to-high thrombus burden). Subsequently, 6F JR4.0, JL4.0, JL3.5, AL1.0 or MPA1.0 guide tubes (from Johnson & Johnson, United States) were inserted through the sheath into the target vessel to perform selective pulmonary artery angiography.

During the angiography process, lesions were classified according to the Japanese BPA Study Group’s angiographic classification system (2015) into three types: (1) Reticular lesions: Characterized by a fine, web-like network of intravascular structures (resembling a spider’s web) that disrupt normal blood flow without complete occlusion. (2) Annular lesions: Defined as concentric, ring-shaped stenosis of the vessel wall (analogous to a finger ring), often involving the proximal pulmonary arteries. (3) Partial occlusion lesions: Partial blockage of the vessel lumen with residual patent channel, further subdivided into: Subtotal occlusion (residual lumen <50% of original diameter) and Stenotic lesions (residual lumen ≥50% but with hemodynamically significant narrowing).

This classification guided the selection of intervention strategies. Based on lesion type, thrombus burden, and post-treatment vascular lumen recovery, appropriate guidewires and balloon dilation catheters were chosen. The Sion, Sion blue, FielderXTR/XT/XTA, Gaia I, Gaia II, Gaia III, Conquest, and Conquest pro guidewires with a diameter of 0.014 inches (produced by Japan ASAHI Company) were inserted through the lesion site into the IKAZUCHI balloon dilation catheters with diameters ranging from 1.2 to 5.0 mm and the Empria balloon dilation catheters (produced by Johnson & Johnson, United States). Based on the thrombus load, the recovery of vascular lumen stenosis after treatment, a 0.035-inch ultra-smooth extended guidewire (Asahi Indak Corporation of Japan) was replaced and inserted into an 8.0–10.0 mm COOK balloon dilation catheter (COOK Company of the United States) if necessary. The expansion was performed using 4 to 14 atmospheres of pressure, with each expansion lasting for 6 to 30 s. The surgery was stopped when the total radiation dose for a single procedure did not exceed 2,000 mGy or when the contrast agent reached 300 mL.

During each BPA treatment session, the following operational details were meticulously recorded: The total amount of contrast agent used by the patient in each surgery was accurately recorded, which ranged from 100 to 300 milliliters. Based on the patient’s renal function and the requirements for lesion display, the dosage of the contrast agent was adjusted reasonably to ensure image quality while minimizing potential damage to the patient’s kidneys. Real-time monitoring and recording of the total radiation dose for each individual surgery were carried out to ensure that it did not exceed 2000 millisieverts. By optimizing imaging parameters and reducing unnecessary exposure time, the radiation dose was effectively controlled, thereby reducing the risk of radiation exposure for patients. The entire operation time from the start of disinfection and draping to the end of the surgery was recorded, which was generally between 60 and 180 min.

### Observation indicators

The WHO pulmonary hypertension functional classification was evaluated before BPA and 3 months after the last BPA (17), including Grade I: The patient had no limitations during physical activities, and such daily physical activities never led to an aggravation of symptoms containing shortness of breath, fatigue, chest pain, or near fainting; Grade II: The patient had mild limitations during physical activities, and there were no discomfort symptoms at rest, but daily physical activities could lead to symptoms containing shortness of breath, fatigue, chest pain, or near fainting; Grade III: The patient had significant limitations during physical activities, and there were no discomfort symptoms at rest, but any physical activity below daily activities could cause an increase in shortness of breath, fatigue, chest pain, or near fainting; Grade IV: The patient was unable to perform any physical activity, and already showed signs of right heart failure at rest, manifested by shortness of breath and fatigue, and any physical activity would worsen the symptoms.

Right heart catheterization parameters including mean pulmonary artery pressure (mPAP) and pulmonary vascular resistance (PVR) were measured using right heart catheterization measurement, and cardiac index (CI) were measured using the indirect Fick method.

The tricuspid annular plane systolic excursion (TAPSE), right ventricular fractional area change (RVFAC), peak velocity of tricuspid regurgitation (TRV), end-diastolic volume (EDV) of the left ventricle, end-systolic volume (ESV) of the left ventricle, and left ventricular ejection fraction (LVEF) were measured using the HD15 color Doppler ultrasound instrument.

Before BPA and 3 months after the last BPA, 3 mL of fasting venous blood was obtained from the patients in the morning. The NT-proBNP level was detected using the chemiluminescence method. The levels of tumor necrosis factor-*α* (TNF-α) and interleukin 6 (IL-6) were detected by means of the enzyme linked immunosorbent assay.

The exercise tolerance was assessed by 6-min walking distance (6-MWD) (18). A 30-meter quiet and straight corridor was selected, and the patient was asked to walk back and forth repeatedly. The 6-min walking distance of the patient was recorded.

Before BPA and 3 months after the last BPA, the forced expiratory volume in 1 s (FEV1) and forced vital capacity (FVC) were measured using a pulmonary function detector, and the FEV1/FVC was calculated.

Before BPA and 3 months after the last BPA, the levels of pulmonary artery systolic pressure and pulmonary artery diastolic pressure were measured using a fully digital color Doppler ultrasound diagnostic instrument.

Before BPA and 3 months after the last BPA, 4 mL of radial artery blood was collected from each patient. The partial arterial pressure of carbon dioxide (PaCO_2_) and partial arterial pressure of oxygen (PaO_2_) were measured by means of the GEM3500 blood gas analyzer (Woffen Company, United States).

The BPA-related complications mainly included pulmonary vascular injury and reperfusion pulmonary edema. Pulmonary vascular injury often occurs during the operation with symptoms such as hemoptysis, coughing, and chest pain. Reperfusion pulmonary edema mainly occurs 24 to 48 h after the operation, presenting as new patchy shadows at the surgical site on chest X-ray or chest CT, accompanied or not by a decrease in blood oxygen saturation or coughing up large amounts of frothy sputum.

### Statistical analysis

All data from this study underwent analysis utilizing GraphPad Prism 10 statistical software. For counting data, the results were presented as (n, %), and the differences between the two groups were assessed using the χ^2^ test. Normality test was performed with Shapiro–Wilk test. Quantitative variables with a normal distribution are expressed as means ± standard deviations (Mean ± SD). In cases where the data exhibit a non-normal distribution, the median and interquartile range [Median (IQR)] are utilized for data representation. For comparison between two independent groups, independent sample Student’s t-test was used for continuous variables with normal distribution, and Mann–Whitney U test was used for variables with non-normal distribution. Effect sizes were calculated and reported with 95% confidence intervals (95% CIs). Statistical significance was defined as *p* < 0.05.

## Results

### Clinical data of patients

This study collected 100 patients with CTEPH underwent BPA treatment, including 52 males and 48 females, with an average age of (62.43 ± 10.59) years. There were 20 patients with a history of hypertension, 15 patients with a history of diabetes, 8 patients with a history of drinking, as well as 5 patients with a history of smoking. Additionally, all 100 patients underwent anticoagulant thrombolytic therapy and pulmonary artery targeted therapy (Table 1).

**Table 1:** Clinical data of patients.

Items	Data
Gender	
Male	52 (52.00)
Female	48 (48.00)
Age (years)	62.43 ± 10.59
BMI (kg/m^2^)	23.85 ± 2.31
History of diabetes	20 (20.00)
History of hypertension	15 (15.00)
Drinking	8 (8.00)
Smoking	5 (5.00)
Anticoagulant thrombolytic therapy	
Rivaroxaban tablet	53 (53.00)
Dabigatran Etexilate Capsules	34 (34.00)
Warevan tablet	13 (13.00)
Pulmonary artery targeted therapy	
Riociguat	19 (19.00)
ERA	16 (16.00)
PDE5i	65 (65.00)

### WHO cardiac function classification

Before BPA, there were 2 cases with Grade I, 52 cases with Grade II, 37 cases with Grade III, as well as 9 cases with Grade IV. Three months after the last BPA, there were 13 cases with Grade I, 68 cases with Grade II, 14 cases with Grade III, as well as 5 cases with Grade IV. Compared with before BPA, the WHO cardiac function classification of Grade I, Grade II and Grade III was significantly improved 3 months after the last BPA (*p* = 0.003, *p* = 0.020, *p* = 0.000; Table 2).

**Table 2:** WHO cardiac function classification.

Time	n	Grade I	Grade II	Grade III	Grade IV
Before BPA	100	2 (2.00)	52 (52.00)	37 (37.00)	9 (9.00)
3 months after the last BPA	100	13 (13.00)	68 (68.00)	14 (14.00)	5 (5.00)
χ^2^		8.721	5.333	13.920	1.229
*p*		0.003	0.020	0.000	0.267
95% CI		0.030–0.551	0.287–0.920	1.796–7.344	0.622–5.149

### Right heart catheterization parameters

**Figure 1:**
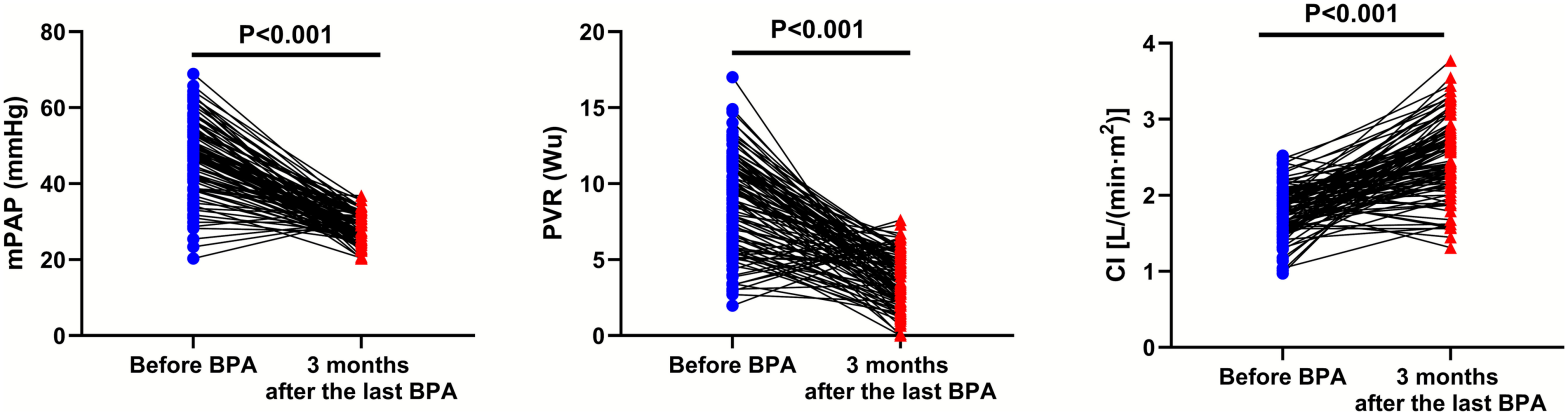
Right heart catheterization parameters before BPA and 3 months after the last BPA.

Compared with before BPA, the mPAP (95% CI: −21.07–−16.92) and PVR (95% CI: −5.832–−4.368) were significantly lower in CTEPH patients 3 months after the last BPA, while the CI (95% CI: 0.590–0.829) was significantly higher in CTEPH patients 3 months after the last BPA (*p* < 0.001; Figure 1).

### Cardiac function indexes

**Figure 2:**
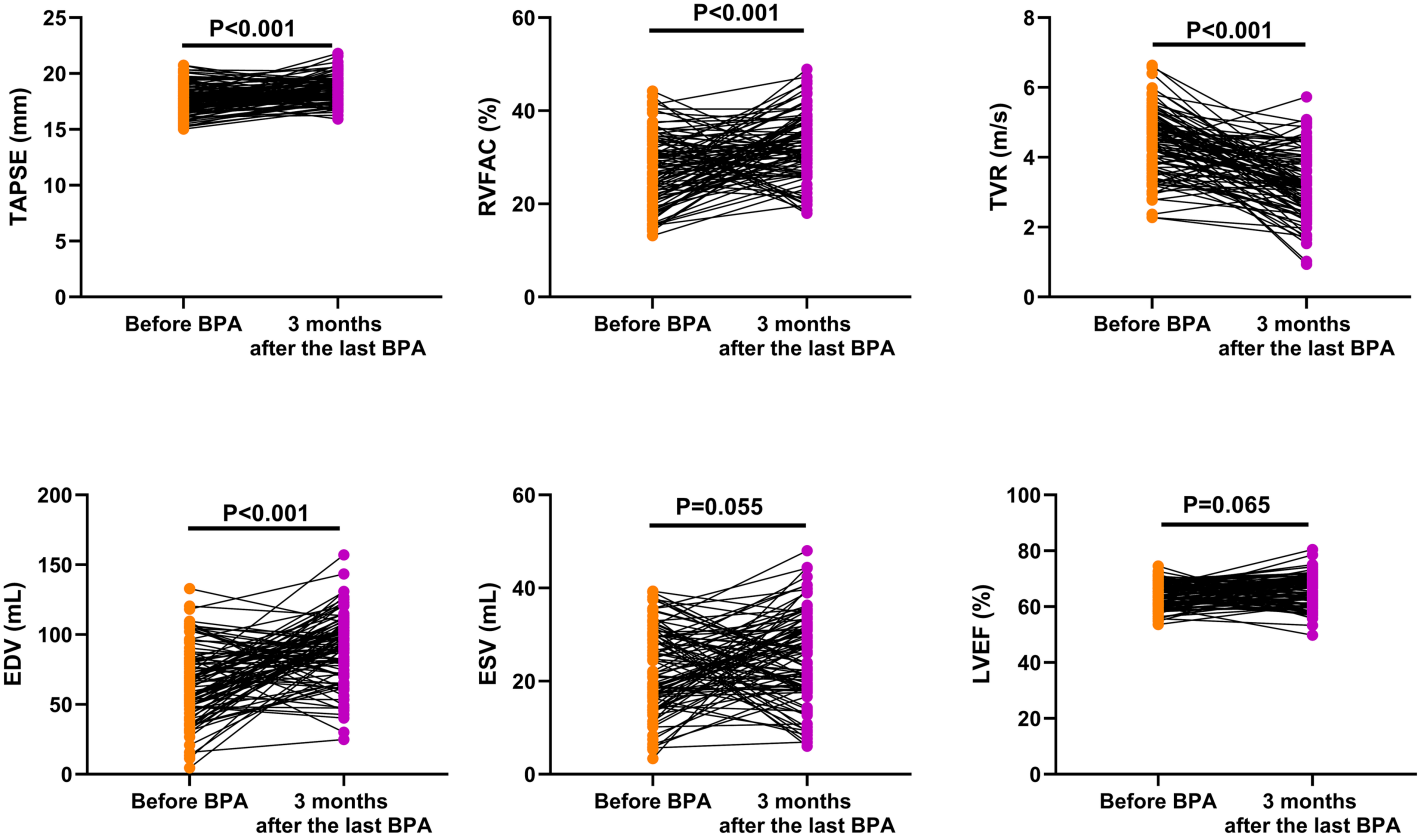
Cardiac function indexes before BPA and 3 months after the last BPA.

Compared with before BPA, the TAPSE (95% CI: 0.543–1.254), RVFAC (95% CI: 2.924–6.936) and EDV (95% CI: −1.451–−0.929) were significantly higher in CTEPH patients 3 months after the last BPA, while the TVR (95% CI: 15.97–30.29) was significantly lower in CTEPH patients 3 months after the last BPA (*p* < 0.001). However, there were no significant differences in ESV (95% CI: −0.062-5.123) and LVEF (95% CI: −0.085–2.748) between before BPA and 3 months after the last BPA (*p* > 0.05), as shown in Figure 2.

### Levels of NT-proBNP, TNF-*α* and IL-6

**Figure 3:**
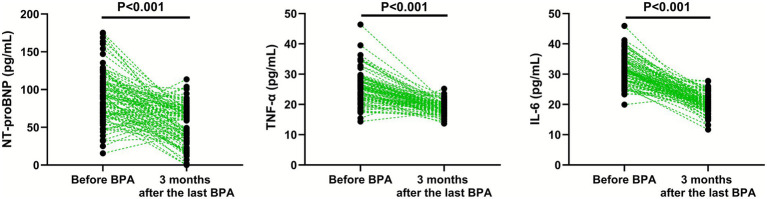
Levels of NT-proBNP, TNF-α, and IL-6 before BPA and 3 months after the last BPA.

Compared with before BPA, the levels of NT-proBNP (95% CI: −46.59–−30.21), TNF-α (95% CI: −8.072–−5.928) and IL-6 (95% CI: −12.02–−9.861) were significantly lower in CTEPH patients 3 months after the last BPA (*p* < 0.001; Figure 3).

### Exercise tolerance

**Figure 4:**
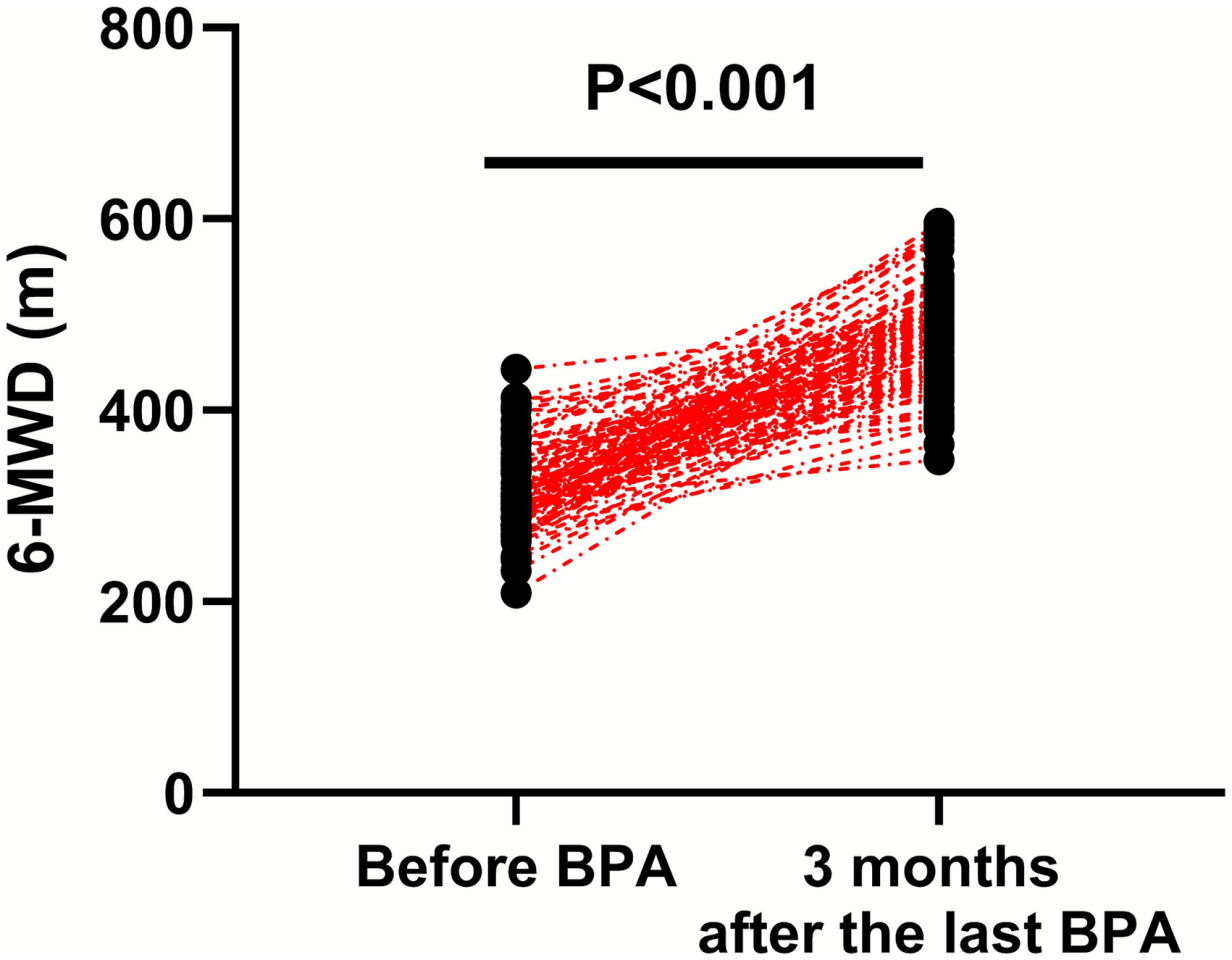
Exercise tolerance before BPA and 3 months after the last BPA.

Compared with before BPA, the 6-MWD was significantly longer in CTEPH patients 3 months after the last BPA (*p* < 0.001, 95% CI: 152.0–178.2; Figure 4).

### Pulmonary function indexes

**Figure 5:**
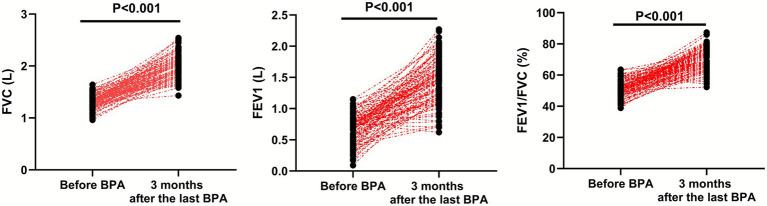
Pulmonary function indexes before BPA and 3 months after the last BPA.

Compared with before BPA, the FVC (95% CI: 0.637–0.742), FEV1 (95% CI: 0.687–0.868) and FEV1/FVC (95% CI: 17.30–20.74) were significantly higher in CTEPH patients 3 months after the last BPA (*p* < 0.001; Figure 5).

### Pulmonary artery pressure

**Figure 6:**
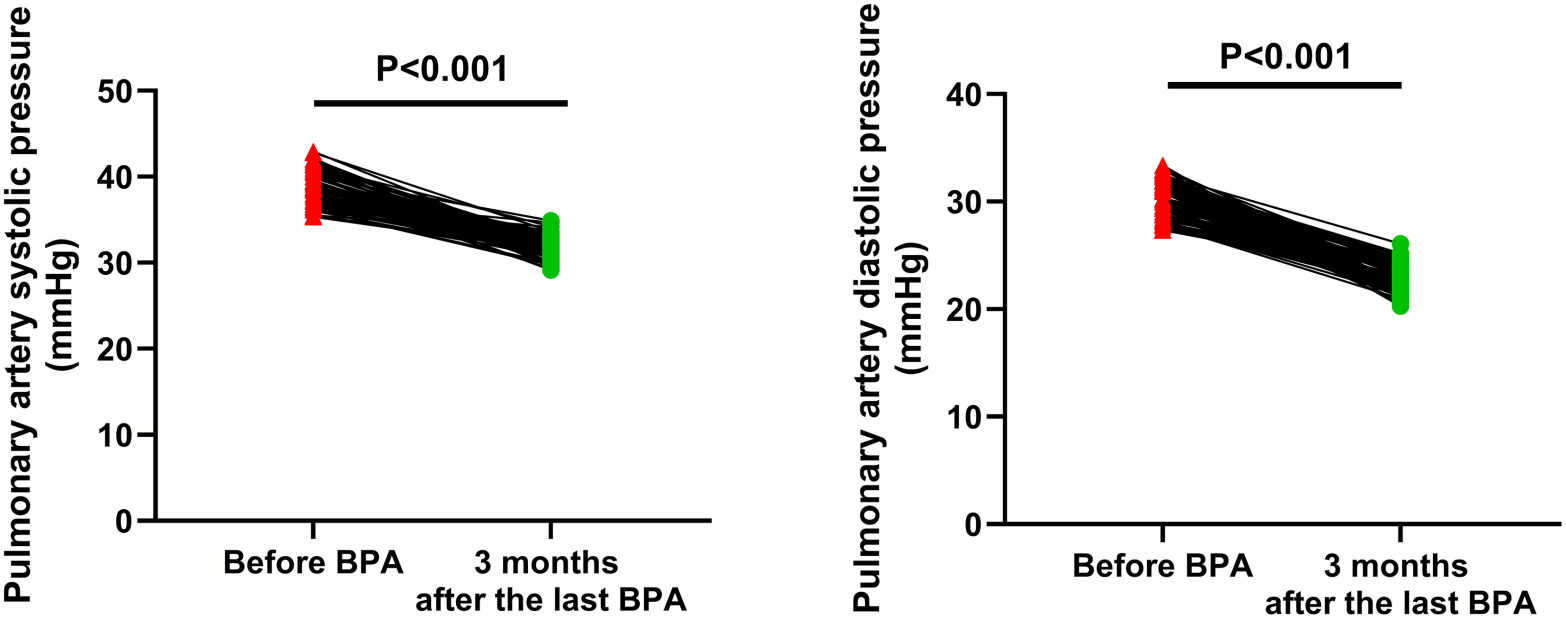
Pulmonary artery pressure before BPA and 3 months after the last BPA.

Compared with before BPA, the levels of pulmonary artery systolic pressure (95% CI: −7.439–−6.581) and pulmonary artery diastolic pressure (95% CI: −8.106–−7.314) were significantly lower 3 months after the last BPA (*p* < 0.001; Figure 6).

### Blood gas indexes

**Figure 7:**
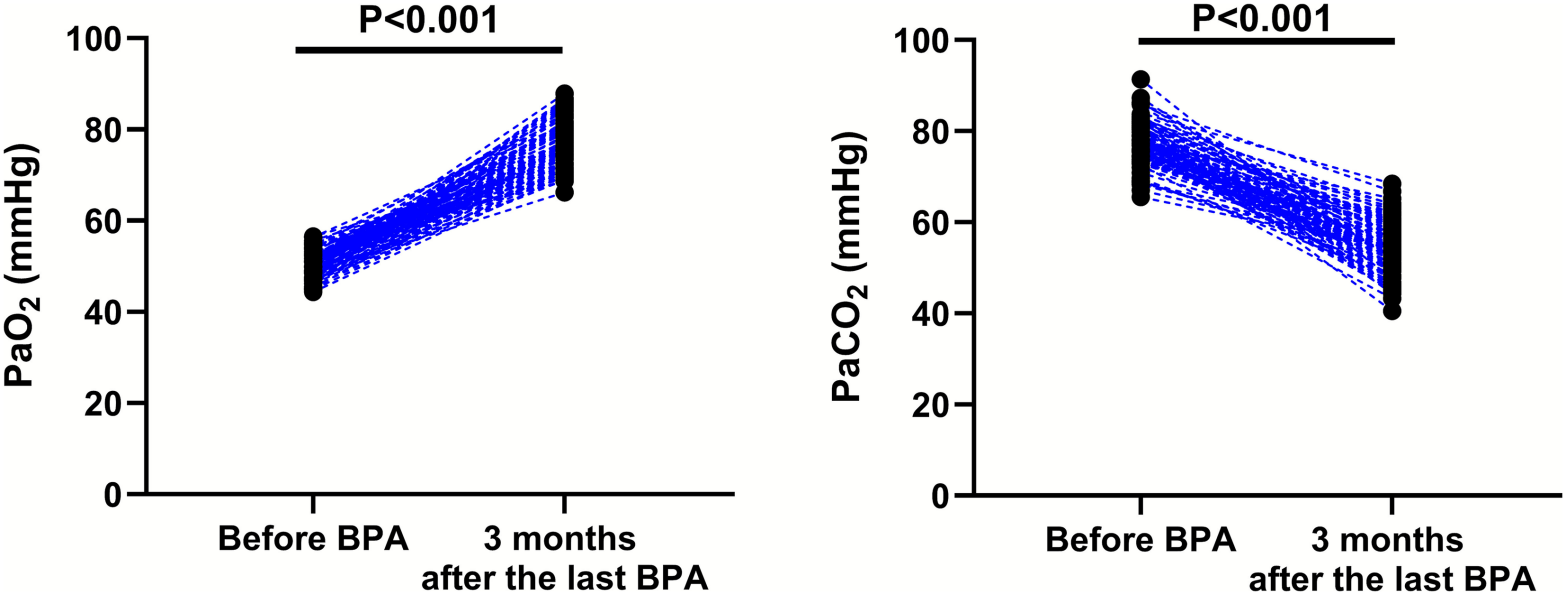
Blood gas indexes before BPA and 3 months after the last BPA.

Compared with before BPA, the level of PaO_2_ (95% CI: 25.06–27.26) was significantly higher while the level of PaCO_2_ (95% CI: −24.71–−21.89) was significantly lower 3 months after the last BPA (*p* < 0.001; Figure 7).

### BPA-related complications

There were 5 cases of hemoptysis, 1 case of reperfusion pulmonary edema, and no other complications occurred.

## Discussion

BPA is an emerging interventional surgical technique for CTEPH that has been developed in recent years (19). It typically employs smaller balloons and a staged, sequential approach to gradually expand the pulmonary artery in the area affected by thrombus embolism, allowing for re-establishment of the pulmonary artery and improvement of blood perfusion (20). The surgical outcomes and safety of this technique have gradually gained recognition from clinical centers both domestically and internationally (21). In recent years, meta-analyses from abroad and reports from several major medical centers in China have successively demonstrated the effectiveness and safety of BPA (22, 23). However, the overall situation in our country is that the implementation of BPA surgeries has been short in duration, started late, and lacks sufficient experience and systematic research.

Our study explored the clinical efficacy of BPA in CTEPH patients, and found that compared with before BPA, the WHO cardiac function classification of Grade I, Grade II and Grade III was significantly improved 3 months after the last BPA, the TAPSE, RVFAC and EDV were significantly higher, the TVR was significantly lower, the 6-MWD was significantly longer, and the FVC, FEV1 and FEV1/FVC were significantly higher. These results implied that BPA could improve the cardiopulmonary function of CTEPH patients. Consistently, Jansa et al. (24) showed significant improvements in New York Heart Association functional class and 6MWD in CTEPH patients underwent BPA. Umemoto et al. (25) found that in 70 patients with CTEPH who underwent BPA, the pulmonary arterial compliance significantly increased and the pulmonary vascular resistance significantly decreased. Additionally, the increase in 6MWD after BPA surgery was related to the increase in pulmonary artery compliance, suggesting that BPA could enhance the exercise tolerance of such patients. Jin et al. (26) discovered that WHO functional class, 6MWD, and cardiopulmonary function were all significantly improved in CTEPH patients after a single BPA session.

Besides, our study also found that compared with before BPA, the mPAP, PVR, the pulmonary artery systolic pressure and pulmonary artery diastolic pressure were significantly lower and the CI was significantly higher 3 months after the last BPA. In line with our results, Kennedy et al. (27) conducted a meta-analysis and discovered that mPAP declined by 13.2 mmHg after BPA surgery. Additionally, Brenot et al. (28) discovered that the mPAP and PVR of CTEPH patients treated with BPA decreased by 22 and 37%, respectively. The observed reduction in mPAP (19 mmHg) and PVR (5.1 Wu) in this study was within a similar range, suggesting that our technical approach has achieved comparable immediate hemodynamic improvement effects. However, compared with the large multi-center data from Europe, the improvement we achieved might be at the medium or slightly higher/lower level within their reported range (29). This difference might be related to the baseline disease severity of the patients, the distribution of lesion types, the surgical staging strategy, and the surgeon’s experience. Future studies need to be conducted with larger samples to refine the analysis and clarify the efficacy characteristics of the specific patient population in our country.

Inflammatory responses are involved in the development of CTEPH (30). TNF-*α* and IL-6 are crucial pro-inflammatory factors that play a role in mediating the progression of various inflammatory diseases (31). NT-proBNP is an important indicator reflecting cardiac function, and it is associated with abnormal hemorheology, ventricular remodeling and heart failure (32). The results of our study indicated that compared with before BPA, the levels of NT-proBNP, TNF-α as well as IL-6 were significantly lower in CTEPH patients 3 months after the last BPA. All these results suggested that BPA could significantly reduce the inflammatory response and improve the degree of heart failure in CTEPH patients. In accordance with our findings, Wiedenroth et al. evaluated the real-life efficacy of BPA procedures in CTEPH patients included in the Greek Pulmonary Hypertension Registry, and discovered that NT-proBNP was decreased by >70% after BPA (33). Magoń et al. suggested that patients with inoperable CTEPH exhibited increased systemic inflammation and endothelial dysfunction, which improved after completion of the BPA treatment (34).

In addition, our study demonstrated that compared with before BPA, the PaO_2_ level was significantly higher while the PaCO_2_ level was significantly lower 3 months after the last BPA. All these results implied that BPA could improve the blood gas level of CTEPH patients. Similarly, Blanquez-Nadal et al. proposed that in CTEPH patients, the levels of PaCO_2_, end-tidal PCO_2_ (PETCO_2_), V̇E and CO_2_output (V̇CO_2_) were significantly improved after BPA (35).

In terms of complications, 5 cases of intraoperative hemoptysis (5.00%) and 1 case of postoperative reperfusion pulmonary edema (1.00%) in this study were all alleviated after close observation or corresponding treatment, and there were no fatal complications. Similarly, a review indicates that with the advancement of technology (such as the widespread use of small balloons and staged treatment strategies) and the accumulation of experience, the incidence of severe complications related to BPA has significantly decreased (36). In addition, a study proposed by Tao et al. suggested that hemoptysis occurred in 5 sessions (7.5%) and reperfusion pulmonary edema (RPE) occurred in 2 sessions (1.5%) in CTEPH patients undergoing BPA (37). However, in a multicenter registry study proposed by Ogawa et al. (38), the incidence rate of hemoptysis was approximately 14.0%, the incidence rate of pulmonary injury was 17.8%, and an incidence rate of pulmonary artery perforation was 2.9%. The complication rate in our study was lower than the above figures, which may be attributed to our meticulous attention to surgical details and the optimization of perioperative management. This provides a practical confirmation that BPA has good safety under strict operational procedures.

This study has several limitations: Firstly, this study is a single-center study with a relatively small sample size. As clinical work continues to deepen and develop, multi-center and prospective studies can be conducted in the future to provide more reliable clinical evidence. Secondly, our study lacks of long-term follow-up data. The follow-up period needs to be extended to further clarify the long-term efficacy of BPA. Thirdly, there is a lack of relevant information regarding pulmonary artery targeted therapy and anticoagulation therapy in this study. Due to the absence of detailed records on the specific details of pulmonary artery targeted therapy and anticoagulation treatment (including the duration of medication, dosage, and duration of treatment), we are unable to conduct an in-depth analysis of the precise interaction mechanisms between these supplementary treatment methods and BPA therapy, nor can we accurately assess their potential impact on the research results. This has led to certain uncertainties in our interpretation of the research results, and we cannot completely rule out the interference of these supplementary treatment factors on the efficacy of BPA therapy. In future studies, we will meticulously document the patients’ medication usage, including the timing, dosage, and duration of the medication. This will enable us to conduct more precise subgroup analyses and further clarify the impact of various factors on the treatment outcome.

## Conclusion

BPA can improve the exercise tolerance, cardiac and pulmonary functions, and blood gas level of patients with CTEPH, and the incidence of complications is relatively low. The results of this study have confirmed that BPA is an effective and relatively safe interventional treatment option for patients with CTEPH. However, to more accurately determine the level and characteristics of BPA treatment in our country, future studies must be designed rigorously, involve multiple centers, and conduct long-term follow-up. Such studies should also be reported in a more systematic and quantitative manner, comparing with global data, in order to clarify advantages, identify gaps, and guide directions.

## Data Availability

The datasets presented in this study can be found in online repositories. The names of the repository/repositories and accession number(s) can be found in the article/supplementary material.

## References

[1] Lang IM Campean IA Sadushi-Kolici R Badr-Eslam R Gerges C Skoro-Sajer N. Chronic thromboembolic disease and chronic thromboembolic pulmonary hypertension. Clin Chest Med. (2021) 42:81–90. doi: 10.1016/j.ccm.2020.11.014, 33541619

[2] Teerapuncharoen K Bag R. Chronic thromboembolic pulmonary hypertension. Lung. (2022) 200:283–99. doi: 10.1007/s00408-022-00539-w, 35643802

[3] Lang I Meyer BC Ogo T Matsubara H Kurzyna M Ghofrani HA . Balloon pulmonary angioplasty in chronic thromboembolic pulmonary hypertension. Eur Respir Rev. (2017) 26:160119. doi: 10.1183/16000617.0119-2016, 28356406 PMC9489135

[4] Matusov Y Singh I Yu YR Chun HJ Maron BA Tapson VF . Chronic thromboembolic pulmonary hypertension: the bedside. Curr Cardiol Rep. (2021) 23:147. doi: 10.1007/s11886-021-01573-5, 34410530 PMC8375459

[5] Hoole SP Jenkins DP. Chronic thromboembolic pulmonary hypertension: interventional approaches. Heart. (2020) 106:1525–31. doi: 10.1136/heartjnl-2019-316291, 32404404

[6] Delcroix M de Perrot M Jaïs X Jenkins DP Lang IM Matsubara H . Chronic thromboembolic pulmonary hypertension: realising the potential of multimodal management. Lancet Respir Med. (2023) 11:836–50. doi: 10.1016/S2213-2600(23)00292-8, 37591299

[7] Jenkins D Madani M Fadel E D'Armini AM Mayer E. Pulmonary endarterectomy in the management of chronic thromboembolic pulmonary hypertension. Eur Respir Rev. (2017) 26:160111. doi: 10.1183/16000617.0111-2016, 28298388 PMC9489144

[8] Pepke-Zaba J Delcroix M Lang I Mayer E Jansa P Ambroz D . Chronic thromboembolic pulmonary hypertension (CTEPH): results from an international prospective registry. Circulation. (2011) 124:1973–81. doi: 10.1161/CIRCULATIONAHA.110.015008, 21969018

[9] Freed DH Thomson BM Berman M Tsui SS Dunning J Sheares KK . Survival after pulmonary thromboendarterectomy: effect of residual pulmonary hypertension. J Thorac Cardiovasc Surg. (2011) 141:383–7. doi: 10.1016/j.jtcvs.2009.12.056, 20471039

[10] Galiè N Humbert M Vachiery JL Gibbs S Lang I Torbicki A . 2015 ESC/ERS guidelines for the diagnosis and treatment of pulmonary hypertension: the joint task force for the diagnosis and treatment of pulmonary hypertension of the European Society of Cardiology (ESC) and the European Respiratory Society (ERS): endorsed by: Association for European Paediatric and Congenital Cardiology (AEPC), International Society for Heart and Lung Transplantation (ISHLT). Eur Heart J. (2016) 37:67–119. doi: 10.1093/eurheartj/ehv317, 26320113

[11] Ghofrani HA Simonneau G D'Armini AM Fedullo P Howard LS Jaïs X . Macitentan for the treatment of inoperable chronic thromboembolic pulmonary hypertension (MERIT-1): results from the multicentre, phase 2, randomised, double-blind, placebo-controlled study. Lancet Respir Med. (2024) 12:e21–30. doi: 10.1016/S2213-2600(24)00027-4, 38548406

[12] Hirakawa K Yamamoto E Takashio S Hanatani S Araki S Suzuki S . Balloon pulmonary angioplasty in chronic thromboembolic pulmonary hypertension. Cardiovasc Interv Ther. (2022) 37:60–5. doi: 10.1007/s12928-021-00775-6, 33928528

[13] Kim NH Delcroix M Jenkins DP Channick R Dartevelle P Jansa P . Chronic thromboembolic pulmonary hypertension. J Am Coll Cardiol. (2013) 62:D92–9. 24355646 10.1016/j.jacc.2013.10.024

[14] Ikeda N. Balloon pulmonary angioplasty for chronic thromboembolic pulmonary hypertension. Cardiovasc Interv Ther. (2020) 35:130–41. doi: 10.1007/s12928-019-00637-2, 31873853

[15] Ogo T Fukuda T Tsuji A Fukui S Ueda J Sanda Y . Efficacy and safety of balloon pulmonary angioplasty for chronic thromboembolic pulmonary hypertension guided by cone-beam computed tomography and electrocardiogram-gated area detector computed tomography. Eur J Radiol. (2017) 89:270–6. doi: 10.1016/j.ejrad.2016.12.013, 28034568

[16] Olsson KM Wiedenroth CB Kamp JC Breithecker A Fuge J Krombach GA . Balloon pulmonary angioplasty for inoperable patients with chronic thromboembolic pulmonary hypertension: the initial German experience. Eur Respir J. (2017) 49:1602409. doi: 10.1183/13993003.02409-2016, 28596435

[17] Kovacs G Bartolome S Denton CP Gatzoulis MA Gu S Khanna D . Definition, classification and diagnosis of pulmonary hypertension. Eur Respir J. (2024) 64:2401324. doi: 10.1183/13993003.01324-2024, 39209475 PMC11533989

[18] Douwes JM Hegeman AK van der Krieke MB Roofthooft MT Hillege HL Berger RM. Six-minute walking distance and decrease in oxygen saturation during the six-minute walk test in pediatric pulmonary arterial hypertension. Int J Cardiol. (2016) 202:34–9. doi: 10.1016/j.ijcard.2015.08.155, 26386916

[19] Yang J Madani MM Mahmud E Kim NH. Evaluation and Management of Chronic Thromboembolic Pulmonary Hypertension. Chest. (2023) 164:490–502. doi: 10.1016/j.chest.2023.03.029, 36990148 PMC10410247

[20] Jaïs X Brenot P Bouvaist H Jevnikar M Canuet M Chabanne C . Balloon pulmonary angioplasty versus riociguat for the treatment of inoperable chronic thromboembolic pulmonary hypertension (RACE): a multicentre, phase 3, open-label, randomised controlled trial and ancillary follow-up study. Lancet Respir Med. (2022) 10:961–71. doi: 10.1016/S2213-2600(22)00214-4, 35926542

[21] Kawakami T Matsubara H Shinke T Abe K Kohsaka S Hosokawa K . Balloon pulmonary angioplasty versus riociguat in inoperable chronic thromboembolic pulmonary hypertension (MR BPA): an open-label, randomised controlled trial. Lancet Respir Med. (2022) 10:949–60. doi: 10.1016/S2213-2600(22)00171-0, 35926544

[22] Zoppellaro G Badawy MR Squizzato A Denas G Tarantini G Pengo V. Balloon pulmonary angioplasty in patients with chronic thromboembolic pulmonary hypertension - a systematic review and Meta-analysis. Circ J. (2019) 83:1660–7. doi: 10.1253/circj.CJ-19-0161, 31231116

[23] Zhang L Bai Y Yan P He T Liu B Wu S . Balloon pulmonary angioplasty vs. pulmonary endarterectomy in patients with chronic thromboembolic pulmonary hypertension: a systematic review and meta-analysis. Heart Fail Rev. (2021) 26:897–917. doi: 10.1007/s10741-020-10070-w, 33544306

[24] Jansa P Heller S Svoboda M Pad'our M Ambrož D Dytrych V . Balloon pulmonary angioplasty in patients with chronic thromboembolic pulmonary hypertension: impact on clinical and hemodynamic parameters, quality of life and risk profile. J Clin Med. (2020) 9. doi: 10.3390/jcm9113608, 33182415 PMC7697583

[25] Umemoto S Abe K Hosokawa K Horimoto K Saku K Sakamoto T . Increased pulmonary arterial compliance after balloon pulmonary angioplasty predicts exercise tolerance improvement in inoperable CTEPH patients with lower pulmonary arterial pressure. Heart Lung. (2022) 52:8–15. doi: 10.1016/j.hrtlng.2021.11.003, 34801772

[26] Jin Q Luo Q Yang T Zeng Q Yu X Yan L . Improved hemodynamics and cardiopulmonary function in patients with inoperable chronic thromboembolic pulmonary hypertension after balloon pulmonary angioplasty. Respir Res. (2019) 20:250. doi: 10.1186/s12931-019-1211-y, 31703589 PMC6842206

[27] Kennedy MK Kennedy SA Tan KT de Perrot M Bassett P McInnis MC . Balloon pulmonary angioplasty for chronic thromboembolic pulmonary hypertension: a systematic review and meta-analysis. Cardiovasc Intervent Radiol. (2023) 46:5–18. doi: 10.1007/s00270-022-03323-8, 36474104

[28] Brenot P Jaïs X Taniguchi Y Garcia Alonso C Gerardin B Mussot S . French experience of balloon pulmonary angioplasty for chronic thromboembolic pulmonary hypertension. Eur Respir J. (2019) 53:1802095. doi: 10.1183/13993003.02095-2018, 31023842 PMC6853610

[29] Delcroix M Pepke-Zaba J D'Armini AM Fadel E Guth S Hoole SP . Worldwide CTEPH registry: long-term outcomes with pulmonary endarterectomy, balloon pulmonary angioplasty, and medical therapy. Circulation. (2024) 150:1354–65. 39286890 10.1161/CIRCULATIONAHA.124.068610 PMC11562489

[30] Li S Gao L Zhang S Zhao Q Yang T Duan A . Association of Systemic Inflammatory Response Index with disease severity and adverse outcome in chronic thromboembolic pulmonary hypertension. J Inflamm Res. (2025) 18:8217–31. doi: 10.2147/JIR.S517285, 40567587 PMC12191145

[31] Elgellaie A Thomas SJ Kaelle J Bartschi J Larkin T. Pro-inflammatory cytokines IL-1α, IL-6 and TNF-α in major depressive disorder: sex-specific associations with psychological symptoms. Eur J Neurosci. (2023) 57:1913–28. doi: 10.1111/ejn.15992, 37070163

[32] Cunningham JW Myhre PL. NT-proBNP response to heart failure therapies: an imperfect surrogate. J Am Coll Cardiol. (2021) 78:1333–6. doi: 10.1016/j.jacc.2021.07.045, 34556319

[33] Karyofyllis P Demerouti E Giannakoulas G Anthi A Arvanitaki A Athanassopoulos G . Balloon pulmonary angioplasty in patients with chronic thromboembolic pulmonary hypertension in Greece: data from the Hellenic pulmonary hypertension registry. J Clin Med. (2022) 11. doi: 10.3390/jcm11082211, 35456303 PMC9028480

[34] Magoń W Stępniewski J Waligóra M Jonas K Przybylski R Podolec P . Changes in inflammatory markers in patients with chronic thromboembolic pulmonary hypertension treated with balloon pulmonary angioplasty. Cells. (2022) 11. doi: 10.3390/cells11091491, 35563797 PMC9102042

[35] Blanquez-Nadal M Piliero N Guillien A Salvat M Thony F Augier C . Neural respiratory drive in chronic thromboembolic pulmonary hypertension: effect of balloon pulmonary angioplasty. Respir Physiol Neurobiol. (2022) 299:103857. doi: 10.1016/j.resp.2022.103857, 35121103

[36] Elhage Hassan M Vinales J Perkins S Sandesara P Aggarwal V Jaber WA. Pathogenesis, diagnosis, and Management of Chronic Thromboembolic Pulmonary Hypertension. Interv Cardiol Clin. (2023) 12:e37–49. doi: 10.1016/j.iccl.2024.04.003, 38964822

[37] Tao XC Peng WH Xie WM Wan J Liu M Gao L . Efficacy and safety of balloon pulmonary angioplasty for chronic thromboembolic pulmonary hypertension. Zhonghua Yi Xue Za Zhi. (2020) 100:437–41. doi: 10.3760/cma.j.issn.0376-2491.2020.06.008, 32146766

[38] Ogawa A Satoh T Fukuda T Sugimura K Fukumoto Y Emoto N . Balloon pulmonary angioplasty for chronic thromboembolic pulmonary hypertension: results of a multicenter registry. Circ Cardiovasc Qual Outcomes. (2017) 10. doi: 10.1161/CIRCOUTCOMES.117.004029, 29101270

